# RNA Accessibility in cubic time

**DOI:** 10.1186/1748-7188-6-3

**Published:** 2011-03-09

**Authors:** Stephan H Bernhart, Ullrike Mückstein, Ivo L Hofacker

**Affiliations:** 1 Theoretical Biochemistry group, Institute for theoretical chemistry, University of Vienna, Währingerstrasse 17, Vienna, Austria

## Abstract

**Background:**

The accessibility of RNA binding motifs controls the efficacy of many biological processes. Examples are the binding of miRNA, siRNA or bacterial sRNA to their respective targets. Similarly, the accessibility of the Shine-Dalgarno sequence is essential for translation to start in prokaryotes. Furthermore, many classes of RNA binding proteins require the binding site to be single-stranded.

**Results:**

We introduce a way to compute the accessibility of all intervals within an RNA sequence in (*n*^3^) time. This improves on previous implementations where only intervals of one defined length were computed in the same time. While the algorithm is in the same efficiency class as sampling approaches, the results, especially if the probabilities get small, are much more exact.

**Conclusions:**

Our algorithm significantly speeds up methods for the prediction of RNA-RNA interactions and other applications that require the accessibility of RNA molecules. The algorithm is already available in the program RNAplfold of the ViennaRNA package.

## Background

The importance of RNA within living cells has been realized in the last two decades. RNA provides a layer of regulation in eukaryotes, e.g. via miRNA, but also in prokaryotes via small RNAs (sRNAs) and riboswitches. Many of these regulatory functions are mediated by RNA interactions. These interactions are mainly realized through Watson-Crick or wobble base pairing between two RNA molecules. For the initialization of these interactions, a part of the interacting molecules has to be single-stranded. The tendency to be single-stranded is thus also important for the quality of putative target sites of miRNAs [1], siRNAs [2] and most probably sRNAs. Furthermore, the accessibilities of the Shine-Dalgarno sequence and the start codons are indicators of translational efficacy [3]. In addition, RNA accessibility will also influence the efficacy of single-strand binding proteins like *HuR *[4]. As it is not known how big exactly a putative target site is, and where it is located, it is best to know the accessibilities of all possible intervals within a RNA molecule. In particular, programs like RNAup [5,6] or IntaRNA [7] predict RNA-RNA interactions by computing a total binding energy *δG*_tot _= *δG*_int _+ *δG*_open_, composed of a stabilizing energy for the intermolecular duplex *δG*_int _and the cost of opening the binding sites *δG*_open_. The opening energy can be computed from the accessibility, defined as the probability *p^u ^*that the binding site is unpaired in equilibrium, via *δG*_open _= -*RT *ln(*p^u^*).

The most naive approach to compute the accessibility of a certain stretch of bases is to use a constrained folding where no base pairs are allowed within a certain stretch of bases and dividing the respective restricted partition function by the unrestricted one. This is done for example in the miRNA target predictor PITA [8]. However, doing this for all *n*^2 ^possible intervals requires (*n*^5^) time. Ding and Lawrence [9] proposed to compute accessibilities by stochastically sampling structures from the Boltzmann ensemble. Sampling structures can be done in (*n*^3^), but necessarily introduces sampling errors which become large if the accessibilities get small, as is necessarily the case for longer regions. In [10], we introduced an algorithm that computes the accessibilities of all intervals of a given length *l *in cubic time. This leads to a (*n*^4^) algorithm when applied to intervals of all possible lengths. In addition, the algorithm could be used as a scanning algorithm that considers only local structures of a maximum length *L *and runs in (*nL*^2^*l*).

Here we introduce an algorithm to compute the accessibilities or single-strandedness of all intervals of an RNA molecule in (*n*^3^) time and (*n*^2^) memory. This is the same complexity as the algorithm to compute the partition function, which is underlying the sampling approaches, like sfold [11], commonly used for this task. However, the probabilities to be accessible for different intervals are not independent. Therefore, if one is interested in more complex questions, e.g. the joint probability that two intervals are accessible, this is no way around sampling the structures of the molecules.

The first predecessor of our algorithm, not yet fully (*n*^3^), has been used in RNAup [5]. The RNAplfold program of the ViennaRNA package [12] originally used the algorithm introduced in [10], but has been rewritten to use the efficient version of the algorithm presented below.

Since our algorithm is based on McCaskill's dynamic programming (DP) algorithm for the partition function [13], we briefly recapitulate the algorithm as implemented in the ViennaRNA package. As usual, we consider only (pseudo)knot-free secondary structures and use the Turner nearest-neighbor energy model [14]. In the inside (forward) recursion, the partition function *Q*(*i*, *j*) of a sequence interval *i*, *j *is split into a part where *i *is unpaired and a part where *i *is paired:(1)

Here, *Q^B ^*(*i*, *j*) is the partition function for a stretch between *i*, *j *given that (*i*, *j*) form a base pair and *Q*(*a*, *b*) = 1 if *a *≥ *b*. A base pair can either close a hairpin loop, an interior loop (including bulge loops and stacks) or a multi(branch) loop.(2)

Here  is short for the Boltzmann factor of a hairpin loop closed by the base pair (*i*, *j*): (, with ),  stands for the Boltzmann factor of the interior loop enclosed by the base pairs (*i*, *j*) and (*k*, *l*). For the multi loop contributions, *Q^M ^*(*i*, *j*) holds the partition function for a part of a multi loop, and *Q*^*M*1^(*i*, *j*) are multi loop contributions that contain exactly one stem, where *i *belongs to the outermost base pair of this stem.

**Figure 1:**
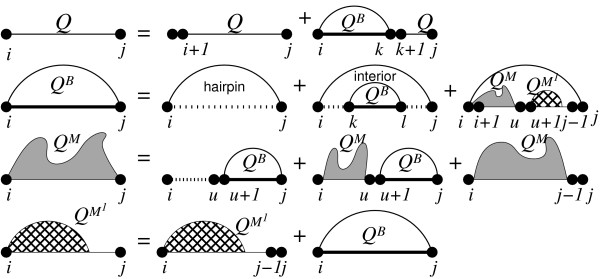
**Partition function folding**. DP decomposition of RNA partition function folding. See text for detailed explanation.

*Q*^*M*1^(*i*, *j*) is needed to keep the multi loop decomposition unambiguous. The factors *a*, *b *and *c *are Boltzmann weighted contributions for closing a multi loop, adding a base pair or an unpaired base to the multi loop, respectively. Note that here, the size of the interior loops is limited to keep the algorithm cubic in time. See Figure 1 for a graphical representation of the recursion.

**Figure 2:**
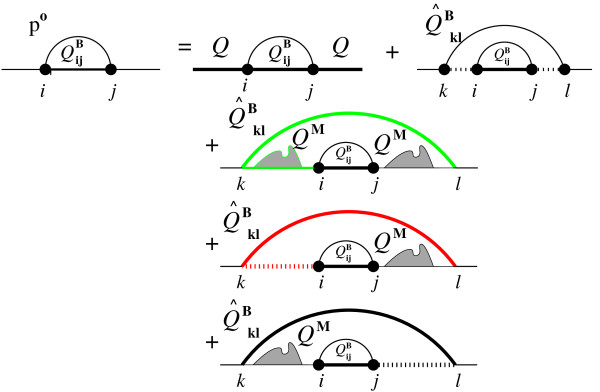
**Pair probability computation**. Computation of pair probabilities. A single base pair (*i*, *j*) either lies within another base pair or not (First term on top). Base pair (*i*, *j*) and the enclosing base pair (*k*, *l*) either form an interior loop (rightmost term on top), or a multi loop. Based on the location of the other components within the multi loop, three possibilities for the closing of a multi loop exist. Here, the contributions collected in auxiliary array  are colored green, in  red.

In the outside (backward) recursion (see Figure 2), the pair probabilities of a RNA molecule of length *n*, that is the quotient between the partition function of all states containing the base pair, *Q^bp^*(*i*, *j*) and the total partition function is computed.(3)

Here, *Q^bp ^*(*i*, *j*) is decomposed into an outer () and an inner (*Q^B ^*(*i*, *j*)) partition function. The computation of the outer partition function of a base pair is split into two cases: The trivial case where no base pair is enclosing the base pair (*i*, *j*), and the case where there exists at least one base pair (*k*, *l*) with *k *<*i *<*j *<*l*.(4)

The interior loop contribution is again kept cubic by the size restriction of the interior loops. However, to keep the multi loop part of the algorithm (*n*^3^), we need to split the double sums over *k *and *l *into two sequential ((*n*)) sums with the help of two auxiliary arrays:

With these arrays, the multi loop part of Eqn 5 becomes:

Each entry of the arrays  and  can be computed in linear time. In fact,  can be computed recursively in constant time. The full probability computation is then:(5)

## Algorithm

**Figure 3:**
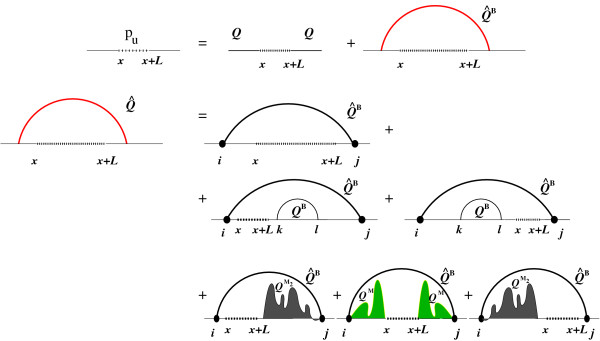
**Unpaired interval computation**. Diagrams of the probability to be unpaired for an interval of length *L *starting at base *x*. The unpaired interval is either enclosed by a base pair or not (top). If enclosed by a base pair, the enclosing base pair can enclose a Hairpin, an Interior Loop or a Multi Loop (rows two to four).

We now extend the McCaskill Algorithm to compute the probabilities *p^u^*(*x*, *x *+ *L*) that a sequence interval *x *... *x *+ *L *contains no paired bases. As can be seen in Figure 3, an unpaired stretch *x *... *x *+ *L *is either not enclosed by a base pair, which again is the trivial case, or there is a base pair (*i*, *j*) enclosing the unpaired region such that *i *<*x *<*x *+ *L *<*j *(we call the sum of these contributions *Q^ub^*(*x*, *x *+ *L*)).

The enclosing base pair (*i*, *j*) can again either close a hairpin, an interior loop or a multi loop.

To keep the computational complexity low, we use decompositions akin to the ones used for the pair probability and partition function computation. Hairpin terms are computed in two steps: First, we consider the case where the 5' base of the enclosing pair is directly adjacent to our region of interest:

Suppose the enclosing pair starts at some position *i *- 1 ≤ *x *- 1, then the 3' end of the pair, *j*, has to be downstream of the unpaired region, i.e. at least at position *x *+ *L *+ 1. This gives rise to a contribution *Q^H^*(*i*, *x *+ *L*). Summing over *i *yields the complete partition function for the hairpin case:(6)

For the interior loop contributions, we do a similar, but a little more complicated decomposition. We note that our region of interest can be located either in the 5' or 3' side of the interior loop and start by computing the partition function *Q*^*I*5^(*i*, *k*) (*Q*^*I*3^(*l*, *j*)) over all structures in which *i *... *k *(*l *... *j*) forms the 5' (3') side of an interior loop (see Figure 3 row 3):

In analogy to *Q^H ^*in the hairpin case, *Q^I ^*(*x*, *x *+ *L*) sums over all cases where the position *x *- 1 preceding our region is paired. The final summation takes care of the cases where the interior loop starts before *x *- 1:

The multi loop contributions can be split into three parts, depending on where within the multi loop the unpaired interval is situated. If the unpaired interval is between the closing base pair and the first stem of the multi loop (i.e. at the 5' side of the multi loop), we compute:

The terms for unpaired intervals that are located between the components of the multi loop and between the closing base pair and the last stem of the multi loop (3' side) are similar to each other:

Here, the contributions  and  stand for the partition function over all partial structures where *x *- 1 is paired with some *g *<*x *- 1, closing a multi loop component (i.e. (*g*, *x *- 1) is one of the interior pairs of the multi loop). The multi loop is closed by a pair (*i*, *j*), where *i *<*g*. The region between *x *... *j *- 1 is a part of the multi loop not yet determined. In the case of , *x *... *j *- 1 has to contain at least one other multi loop component. In , at least one other multi loop component exists between *g *and *i*, so that no additional multi loop component is needed.

Using the matrices from above, it is easy to see that the total computation for a single interval is linear in time:

## Implementation

The matrices needed for the computation of the accessibilities mostly contain terms that are computed anyway during the inside or outside recursions. These matrices can thus be committed to memory with little additional cost. The two contributions *Q*^*I*5 ^and *Q*^*I*3 ^are saved during the computation of the base pair probabilities in Eqn 4. The multi loop terms  and  are also computed during the computation of the pair probabilities in Eqn 5, while *Q*^*M*2 ^is saved during the forward recursion Eqn 2. The computation of the accessibilities is thus conveniently performed after the outside recursion. Some of the matrices needed for the computation of *Q^ub ^*can be computed recursively:

and thus require negligible additional computational costs. Due to the layout of this algorithm, it is easily possible to split the terms and e.g. find the probability of an interval to be within a hairpin loop or an interior loop. This can be useful if special types of RNA interactions, for example kissing hairpin interactions, are to be considered.

### Minimum free energy version

The minimum free energy (MFE) version of this algorithm can be used to compute one optimal structure that has an unpaired stretch *x *... *x *+ *L *for every single interval *x *... *x *+ *L*. In principle, the computation of the optimal energy and the backtracking procedure of its structure are similar to the partition function version described above. Because ambiguity is of no concern, only one matrix for multi loop contributions has to be filled. Thus, after computing the "usual" matrices for RNA minimum free energy prediction (the minimum free energy *F *(*i*, *j*), the minimum energy given that *i *and *j *form a base pair *C*(*i*, *j*), and the minimum free energy for multi loop segments *M *(*i*, *j*)), we need to fill the following matrices:

Here, I is the energy of an interior loop. The minimum energy *f_a _*(*x*, *x *+ *L*) with an accessible interval between *x *and *x *+ *L *is then:

In our current implementation, computation of the structure on the outside of a base pair is done by doubling the sequence, doing a simple cofolding and backtracking *j*, *i *+ *n *for the base pair (*i*, *j*). This leaves room for improvement, as the memory consumption is twice as high as is strictly necessary. Backtracking the *f_a _*(*i*, *j*)s gives the secondary structures to the energies.

**Figure 4:**
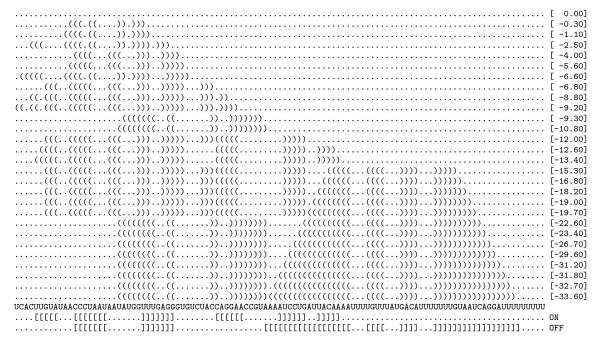
**Structures of a growing RNA molecule**. Minimum free energy structures of the growing chain of the ydhL Adenine riboswitch. The list of all optimal *f_a_*(*i*, *n*) structures is shown. The 3' dots correspond to the unpaired intervals *i *... *n*. The bottom two (square bracketed) structures show the ON and the OFF state of the riboswitch taken from [16]. As can be seen, the ON state corresponds to an intermediate mfe structure (-13.40 kcal/mol, green 5' box), while the terminator loop is part of the overall minimum free energy structure (red 3' box).

As one possible application, the *f_a _*(*i*, *n*) subset of the secondary structures can be viewed as the minimum free energy structures during transcription of a RNA molecule. The unpaired interval in this case is regarded as the part of the molecule that is not yet transcribed. As an example, we show the ydhL Adenine riboswitch in Figure 4.

### Application

The method presented here is currently available in the RNAplfold program of the Vienna RNA package, versions 1.8.x and newer. In addition to global folding the program allows to compute accessibilities for local structures using a sliding window approach. In this case accessibilities for a region are averaged over all sequence windows containing this region. Technically, all that has to be done for the local folding version, is to replace the outside partition function  by averaged versions . The computation of the s has to reflect the fact that sub-sequences of different length will appear in a different number of windows. For example, using a window size *W*, the contribution of  to the computation of , *k *<*i *<*j *<*l*, would be:

The  are subsequently also used in the computation of the accessibilities. This makes the program applicable to even the largest sequences, such as complete chromosomes or all mRNAs of an organism. Several programs, like RNAxs [2], IntaRNA [7], and RNAplex [15] are already using the accessibility computations implemented in RNAplfold with great success to rapidly predict accessibilities of putative target sites on mRNAs.

## Competing interests

The authors declare that they have no competing interests.

## Authors' contributions

UM developed the first versions of the algorithm, SHB developed the current algorithm and integrated it into RNAplfold. ILH initiated and supervised the work. Regrettably, UM died during the preparation of this manuscript. She will always be remembered fondly. ILH and SHB jointly wrote and approved the manuscript.
